# Molecular Morbidity Score–Can MicroRNAs Assess the Burden of Disease?

**DOI:** 10.3390/ijms25158042

**Published:** 2024-07-24

**Authors:** Thomas Butler, Matthew G. Davey, Michael J. Kerin

**Affiliations:** 1 Department of Surgery, Lambe Institute for Translational Research, University of Galway, H91 TK33 Galway, Ireland; matthewdavey21@rcsi.ie (M.G.D.); michael.kerin@universityofgalway.ie (M.J.K.); 2 Department of Surgery, University Hospital Galway, Newcastle Road, H91 YR71 Galway, Ireland

**Keywords:** multimorbidity, comorbidity, microRNA, hallmarks of ageing, prognosis, chronic disease

## Abstract

Multimorbidity refers to the presence of two or more chronic diseases and is associated with adverse outcomes for patients. Factors such as an ageing population have contributed to a rise in prevalence of multimorbidity globally; however, multimorbidity is often neglected in clinical guidelines. This is largely because patients with multimorbidity are systematically excluded from clinical trials. Accordingly, there is an urgent need to develop novel biomarkers and methods of prognostication for this cohort of patients. The hallmarks of ageing are now thought to potentiate the pathogenesis of multimorbidity. MicroRNAs are small, regulatory, noncoding RNAs which have been implicated in the pathogenesis and prognostication of numerous chronic diseases; there is a substantial body of evidence now implicating microRNA dysregulation with the different hallmarks of ageing in the aetiology of chronic diseases. This article proposes using the hallmarks of ageing as a framework to develop a panel of microRNAs to assess the prognostic burden of multimorbidity. This putative molecular morbidity score would have many potential applications, including assessing the efficacy of clinical interventions, informing clinical decision making and facilitating wider inclusion of patients with multimorbidity in clinical trials.

## 1. Introduction

Multimorbidity is a ubiquitous feature of clinical practice and is associated with adverse outcomes for patients, complex challenges for clinicians and increased costs for healthcare systems. Despite a reported global prevalence of at least 40%, which is inexorably increasing, multimorbidity is critically neglected in clinical trials and international guidelines [1]. There is a need to develop novel methods of predicting prognosis for patients with multimorbidity, which could facilitate personalised management strategies. To enable this, research is beginning to focus on identifying biomarkers which could enhance insight into the pathological mechanisms driving multimorbidity and offer innovative clinical utilities. MicroRNAs are small, regulatory, noncoding RNAs which control gene expression and cellular activity and have been implicated in the pathophysiology of many chronic diseases. It is unclear, however, how microRNA expression is associated with the burden of multimorbidity.

## 2. Aims

This study aims to demonstrate the urgent need to develop prognostic biomarkers to assess the burden of multimorbidity, then highlights the putative utility of microRNAs in this area and identifies specific microRNAs for use in future projects.

## 3. Multimorbidity

Comorbidity was originally defined by Feinstein 1970 as a distinct “clinical entity” which occurs in addition to an index disease [2]. Since then, a precise and universally accepted definition has not been agreed upon; it has been suggested that comorbidity can be conceptually divided into diagnostic, therapeutic and prognostic comorbidities which affect the diagnosis, treatment and prognosis of primary conditions, respectively [3]. This makes a crucial distinction between more acute conditions, such as minor musculoskeletal injuries or upper respiratory tract infections, and important chronic diseases which have broader clinical relevance [3]. Studies have demonstrated that comorbidity is associated with critical outcomes such as mortality, length of stay and readmission rates [4].

There is also a subtle distinction between comorbidity and multimorbidity, with the latter considering the co-occurrence of at least two chronic diseases [5] without considering an index disease [6]. Historically, there has been a greater emphasis on comorbidity, particularly in secondary and tertiary care settings, due to a more reductionist, disease-focused model of healthcare and biomedical research [7]. There is growing appreciation, however, of the value of the more wholistic and patient-centred perspective that the concept of multimorbidity offers, with authors acknowledging the false dichotomy between the two [6].

The prevalence of multimorbidity is increasing across the globe, though precise values vary based on the definition and methodology used by studies [5]. A recent systematic review and meta-analysis which looked at 193 studies found that the pooled prevalence of multimorbidity was 42.4%, with higher prevalences in older age groups but no significant difference between high- and low- or middle-income countries [1]. Factors such as improved diagnostic and management strategies for diseases, sedentary lifestyles predisposing to obesity and ageing populations are thought to contribute to this rising prevalence globally [8]. National populational studies have found that multimorbidity is more common in patients from lower socio-economic backgrounds, female patients and patients who smoke [9]; whilst it can occur at any age, multimorbidity is more common amongst older patients, and the number of comorbidities increases with age [9,10]. Moreover, having a single chronic disease at baseline increases the risk of developing subsequent comorbidities in a potentially self-propagating cascade [11]. As well as affecting high-income countries, the burden of chronic diseases has a significant effect on low-income and middle-income countries, with three-quarters of global mortality rates from noncommunicable diseases occurring in these settings [12,13].

This rising prevalence has serious implications for clinical practice. As well conferring the burden of composite diseases, multimorbidity is associated with factors such as increased hospitalisations, fragmentation of care and polypharmacy [5]. This creates more complex healthcare needs for patients and encumbers management for clinicians, which ultimately results in increased costs for healthcare systems [14,15,16]. Accordingly, multimorbidity has been associated with poor clinical outcomes for patients, including impaired physical and mental health functioning, higher mortality rates and frailty [17,18,19].

Despite the prolific effects of multimorbidity on clinical practice, there is a critical oversight of this in guidelines. Most guidelines focus on individual diseases and provide recommendations which are then applied cumulatively to patients with multimorbidity [20]; this is laborious and potentially harmful for patients, as these single-disease guidelines may provide conflicting and potentially contradictory recommendations [8]. There are logistical issues involved with providing recommendations for the many possible combinations of diseases [21], and guidelines are also often constructed using evidence from randomised control trials which have explicitly excluded older patients with comorbidities [8,22]. Indeed, a recent systematic review found that 80% of included randomised controlled trials excluded over half of the prospective patients, largely due to their age, comorbidities and coprescribing [23]. More specifically, clinical trials for heart failure often exclude patients with coronary heart disease, valvular disease and arrhythmias, as well as other commonly occurring and clinically important comorbidities [20]. Indeed, the exclusion of patients with clinically important comorbidities has been demonstrated in RCTs focusing on interventions for many chronic conditions, including stroke, diabetes, chronic obstructive pulmonary disease (COPD) and cancer [22,24]. This significantly limits the clinical applicability of results from these studies.

A National Institute of Clinical Excellence guideline has been developed which focuses on addressing multimorbidity; this recommends a wholistic and patient-centred approach to care which minimises the burden of treatment and disease for patients and optimises the coordination of care whilst proposing methods of identifying patients who have clinically significant multimorbidity [25]. Crucially, this guideline acknowledges the need for further research, particularly addressing issues such as the cost-effectiveness of different strategies for managing multimorbidity, as well developing tools to better predict outcomes for patients with multimorbidity [25]. More recently, authors have reiterated the urgent need for further research in this field, in particular exploring the ability to identify patients with clinically significant multimorbidity by developing superior methods of prognostication [8].

There are numerous tools which currently provide a summative assessment of the burden of multimorbidity for patients to predict clinical outcomes [1]. Sarfati divided these instruments into four categories based on whether they use a simple disease count from a prespecified reference list, assess the functionality of individual organ systems, apply differential weightings to individual conditions based on their clinical significance to generate indices or use alternative methods [26]. For each of these approaches, a list of chronic diseases affecting a patient is compiled and used to generate a prognostic score. A recent systematic review including 96 studies identified 33 different instruments that were used to assess multimorbidity [27]. The authors found that the most commonly used methods for assessing multimorbidity were simple disease counts and weighted disease indices such as the Charlson Comorbidity Index, Elders Risk Assessment and Elixhauser Index, with additional studies using case-mix or pharmaceutical-based strategies [27]. These instruments could successfully predict numerous clinical outcomes, including mortality, quality of life and hospitalisations, which have been identified as critical metrics for multimorbidity research by international consensus [28]. No consideration was given in these studies, however, to the potential use of molecular biomarkers for assessing multimorbidity.

Moreover, there are significant limitations associated with the current approaches. Direct counting methods can be cumbersome and inherently overlook the differences in clinical significance between the individual conditions [29]; weighted indices are often affected by factors such as patient populations, outcomes being investigated and overall context for assessment, which restricts how they can be applied in different clinical settings [27]. There are also significant challenges posed by data collection. Researchers are exploring the relative merits of different approaches including using chart review, discharge notes and administrative information [3]; however, there is currently no gold-standard approach for collecting chronic disease data [27]. Developing a theoretical understanding of comorbidity, including at a molecular level, could facilitate optimised and more generalisable methods of assessment.

Indeed, there has been growing research exploring the aetiological mechanisms of multimorbidity. It is thought that the biochemical hallmarks of ageing and chronic inflammation contribute to the aetiology of chronic diseases and resultant multimorbidity as the pathophysiological effects of these mechanisms accrue [8]. Genomic instability, epigenetic changes, telomere depletion, impaired proteostasis and cell signalling, mitochondrial dysfunction, cellular senescence and stem cell depletion have all been associated with the development of chronic, age-related diseases, including chronic lung diseases, neurodegenerative conditions, musculoskeletal conditions and malignancies [8]. There is a relative paucity of studies investigating how these factors specifically contribute to multimorbidity, but this is a growing area.

For example, Niedzwiedz et al. investigated the effect of telomere length on the incidence of chronic diseases [30]. They found that whilst telomere length was associated with specific chronic diseases, it had very limited associations with multimorbidity [30]. The study was limited by using a narrow set of reference diseases and did not use validated indices for assessing multimorbidity and its potential prognostic significance [30]. A separate study found that shortened absolute telomere length was associated with sarcopenia, frailty and adverse survival outcomes in patients with multimorbidity, though the actual impact on multimorbidity was not assessed [31]. Mitochondrial dysfunction in T cells was associated with secretion of inflammatory cytokines which induce senescence in a murine model of ageing and multimorbidity [32]. This also led to multisystem degeneration, with the mice exhibiting immunosuppression, heart failure, valvular abnormalities, sarcopenia and signs of neurological impairment, with associated changes in messenger RNA expression [32].

Accordingly, the pathophysiology of multimorbidity is intrinsically related to the geroscience hypothesis [8]. This proposes that the general mechanisms of deterioration associated with ageing lead to multisystem disease and so have inherent value as prospective therapeutic and clinical targets [33]. Indeed, a comprehensive literature review, which considered over 900,000 scientific abstracts, identified five hallmarks of ageing which had statistically significant associations with the presence of multimorbidity in patients [34].

Researchers are using this theoretical knowledge to identify clinically useful biomarkers for patients with multimorbidity [8]. Inflammatory and anabolic hormone biomarkers have been associated with higher levels of multimorbidity as assessed by the number of diagnosed diseases [35]. A different study used linear regression methods to identify a panel of 11 cardiovascular protein biomarkers which could predict the burden of multimorbidity out of 80 cardiovascular protein biomarkers included in the study. Interestingly, this panel was used to generate a “biomarker score” which was associated with prognostic outcomes, including mortality [36]. There is also extensive research focusing on how biomarkers can predict clusters of comorbidities, which could be used to target preventative interventions to at-risk populations [37]. There is a need for further studies to explore prospective biomarkers for patients with multimorbidity [38]. There appears to be a particular paucity of studies investigating how biomarkers can be used to assess the burden of multimorbidity by correlation with clinically validated indices.

## 4. MicroRNAs and Multimorbidity

MicroRNAs are small, regulatory, noncoding RNAs which control gene expression and cellular activity and have been implicated in the pathophysiology of many chronic diseases [39]. Consequently, research has shown that microRNA expression can guide the diagnosis, characterisation and prognosis of these conditions [40,41,42,43]. Specific properties enhance the putative utility of microRNA as a clinical biomarker. For example, microRNA is stable in circulation, and fixed samples and can be extracted by liquid biopsy from blood, urine and other bodily fluids [44].

Whilst there is a prodigious area of study investigating the clinical significance of microRNA in specific diseases, the implications of microRNA in the context of multimorbidity is comparatively under-researched. This is reflected in the findings of a historical review paper which found that among 59 papers which assessed the clinical significance of biomolecules in the context of comorbidity, only 5% considered microRNA [45]. At this time, there was growing recognition that molecular markers could be used to classify diseases and predict comorbidity relationships [46]. Subsequently, network models were developed which used microRNA to investigate disease–disease relationships [47]. Studies have found that including noncoding RNA such as microRNA improves the predictive accuracy of network models for disease associations, thus helping to elucidate cluster patterns for comorbidities [48].

Indeed, numerous studies have used microRNAs to explore associations between specific comorbidities. MicroRNAs have been identified as important mediators of the cardiovascular complications of rheumatoid arthritis and other systemic autoimmune disorders [49,50], whilst microRNAs have also been used to explore the mechanisms contributing to nonalcoholic steatohepatitis and periodontitis occurring as comorbidities for patients with psoriasis [51,52]. Similarly, extracellular vesicle-associated microRNAs are thought to contribute to the aetiology of multimorbidity in patients with chronic obstructive pulmonary disease [53]. Fascinating studies are using network medicine approaches, augmented by deep learning algorithms, to predict comorbidities for patients infected with SARS-CoV-2 by looking at microRNA expression profiles and identifying common molecular pathways [54]. In the field of neuropsychiatry, numerous studies have explored how microRNA can be used to predict the occurrence of diseases such as major depression, anxiety and substance use disorder as comorbidities [55,56]. A significant body of research is also investigating the clinical significance of microRNAs relating to obesity and how these can be used to predict metabolic syndrome and other related comorbidities [57,58,59].

There remains, however, a relative paucity of studies investigating how microRNAs can assess the prognostic burden of multimorbidity. This article will now identify specific microRNAs for use in future multimorbidity studies by establishing a panel of microRNAs associated with the hallmarks of ageing.

## 5. MicroRNAs and the Hallmarks of Ageing

The foregoing sections of the article established that the hallmarks of ageing are thought to be critical to the aetiology of multimorbidity. López-Otín et al. first proposed the nine original hallmarks of ageing in 2013 [60] to describe the mechanisms which cause the physiological deterioration and chronic diseases strongly associated with ageing. In 2023, Lopez-Otin et al. proposed three additional hallmarks—disabled macro-autophagy, chronic inflammation and dysbiosis—to account for further advances in the mechanistic theories of ageing [61]. These authors also categorised the expanded twelve hallmarks as primary, antagonistic or integrative, based, respectively, on whether they were definitively pathological, were pathological only in specific circumstances or reflected a failure of homeostatic mechanisms to compensate for accumulated physiological damage [61]. Substantial research has demonstrated how these hallmarks work synergistically to cause chronic age-related diseases [62]. Studies have also elucidated the molecular determinants of these hallmarks and have investigated how nucleic acids such as microRNAs provide transcriptional control [63].

### 5.1. Primary Hallmarks

#### 5.1.1. Genomic Instability

A myriad of deleterious factors, from both endogenous and exogenous sources, contribute to DNA damage, which is exacerbated by impairments in DNA repair networks [61]. This leads to genomic instability, which has been implicated in the pathophysiology of ageing and associated chronic diseases (see Table 1) [61].

Research has explored how microRNA dysregulation contributes to genomic instability and resultant disease [64]. Lämmerhirt et al. recently demonstrated that the loss of miR-101-3p in human melanoma cells confers genomic stability, which prevents apoptosis [65]. miR-101-3p was found to directly target lamin B1, ATP-dependent helicase ATRX, caspase3 and poly(ADP-ribose)-polymerase, which are important regulators of genomic integrity, and the re-expression of miR-101-3p led to an increase in DNA damage and induction of apoptosis [65]. In colorectal cancer cells, Wang et al. found that miR-653-3p inhibited sirtuin 1 (SIRT1) whilst promoting the phosphorylation of signal transducer and activator of transcription protein 3 (STAT3) and promoting Twist family bHLH transcription factor 1 (TWIST1) expression. This contributed to the chromosomal instability and increased DNA damage seen with the ectopic expression of miR-653-3p [66]. Interestingly, genomic instability which is mediated by microRNA dysregulation has been associated with the pathophysiological sequelae of chronic inflammatory conditions. For example, In the ascending colon of patients with primary sclerosing cholangitis, upregulated miR-155 expression levels was associated with high microsatellite instability and the inhibition of suppressors of cytokine signalling 1 (SOCS1), which resulted in STAT-3 induction [67]. Moreover, the absence of miR-155 in the sigmoid colon of these patients activated the interleukin-6 (IL-6)/sphingosine-1-phosphate receptor 1 signalling pathway and affected the IL-17/forkhead box O3 (FOXP3) ratio, which was indicative of chronic inflammation [67].

MicroRNA may also mediate the effects of exogenous factors on genomic stability. Interestingly, dysregulation of the hypoxia-inducible factor-1α/miR-210/RAD52 pathway leading to defects in DNA repair is thought to contribute to genomic instability and mediate the malignant transformation, which occurs secondary to the DNA damage induced by nickel nanoparticles [68]. miR-145 has also been shown to impair the repair of DNA double-strand breaks by impeding the classical nonhomologous end-joining pathway through the suppression of DNA-dependent protein kinase [69]. This led to an increase in DNA damage and hypersensitivity to ionising radiation [69].

Researchers are investigating how microRNAs which are associated with genome instability can be used to predict prognosis for patients. In 2024, Zhang et al. assessed features of genomic instability which were exhibited by patients with breast cancer [70]. As well as finding that genomic instability was associated with adverse outcomes for this cohort, the authors found that the expression levels of miR-105-5p and miR-767-5p were significantly associated with features of genomic instability and could be used to predict prognosis [70]. In a similar study, Xu et al. identified a panel of 12 genome instability microRNAs which generated a microRNA signature which could predict the prognosis of patients with gastric cancer [71].

**Table 1:** MicroRNA and the primary hallmarks of ageing.

Hallmark	MicroRNA	Effect	Model	Target/Mechanism	Findings	Reference
**Genomic Instability**						
	miR-101-3p	↑	Human melanoma cells and melanocytes	Lamin B1, ATRX, CASP3 and PARP	Re-expression of miR-101-3p led to an increase in DNA damage and induction of apoptosis	[65]
	miR-105-5p and miR-767-5p	↑	Patients with breast cancer	Untested	2 microRNA signature was associated with genomic instability and could predict prognosis	[70]
	miR-653-3p	↑	Human colorectal cancer cells	SIRT1/TWIST1 signalling pathway	Ectopic expression of miR-653-3p induced increased DNA damage and chromosomal instability but inhibited apoptosis	[66]
**Telomere Attrition**						
	miR-340-5p	↓	Murine model of Alzheimer’s disease	POT1	miR-340-5p upregulated telomerase activity and increased cellular telomere length, improving Alzheimer’s disease symptoms	[72]
	Deleting miR-126a	↓	Murine model of cholestasis	versican	Deleting miR-126a induced telomere shortening and associated inflammation and hepatic dysfunction	[73]
	miR-185	↑	Human cell lines	POT1	miR-185 reduces P0T1 and overexpression increases telomere dysfunction-induced foci signals and cellular senescence	[74]
**Epigenetic Alterations**						
	miR-148a	↓	Pancreatic surgery specimens	N/A	Hypermethylation of the DNA region encoding miR-148a differentiates chronic pancreatitis and pancreatic ductal adenocarcinoma	[75]
	miR-7	↓	Buccal epithelial samples from patients with COPD	N/A	miR-7 methylated levels could differentiate COPD phenotypes	[76]
	miR-223-3p	↑	Gastric cancer tissue specimens	Arid1a	Mir-2223-3p promotes the progression of gastric cancer	[77]
**Loss of Proteostasis**						
	miR-34	↓	*Drosophilia melanogaster*	H3K27me3, Lst8 subunit of TORC1	Loss of miR-34 expression associated with increased protein accumulation, early ageing and neurodegeneration	[78]
	miR-9	↓	Multiple models for Hutchinson–Gilford progeria syndrome	Lamin A and progerin expression	miR-9 inhibits lamin A and progerin expression in neural cells, mitigating toxic accumulation and protecting against neurodegeneration	[79]
	miR-320a	↓	Colorectal cancer cells	eIF2, unfolded protein response	miR-320a regulates the unfolded protein response in colorectal cancer cells	[80]
**Disabled Macro-Autophagy**						
	miR-33	↑	BAL cells from patients with idiopathic pulmonary fibrosis	Mitochondrial homeostasis and autophagy pathways	Inhibition of miR-33 ameliorates mitochondrial homeostasis and autophagy, decreasing inflammation after bleomycin exposure	[81]
	miR-125b	↑	Thyroid surgical specimens	MAPK and AKT/mTOR signalling	miR125 expression was associated with thyroid cancer invasion and BRAFV600E mutation status	[82]
	miR-494	↑	Rat model of diabetic cardiomyopathy	PI3K/AKT/mTOR pathway	Decreased miR-494 expression reduced apoptosis and autophagy induced by hyperglycaemia	[83]

↑ denotes positive association between microRNA and disease progression/hallmark of ageing; ↓ denotes negative association between microRNA and disease progression/hallmark of ageing; miR, microRNA; ATRX, ATP-dependent helicase ATRX; CASP3, caspase 3; PARP, poly(ADP-ribose)-polymerase; SIRT1, sirtuin 1; TWIST1, Twist family bHLH transcription factor 1; POT1, protection of telomere 1; COPD, chronic obstructive pulmonary disease; TORC1, target of rapamycin complex 1; eIF2, eukaryotic initiation factor 2; BAL, bronchoalveolar lavage; MAPK, mitogen-activated protein kinase; AKT, protein kinase B; mTOR, mechanistic target of rapamycin; PI3K, phosphoinositide 3-kinase; N/A, not available.

#### 5.1.2. Telomere Attrition

The progressive shortening of telomeres occurs following cell division in the absence of telomerase and has been associated with ageing and numerous chronic diseases [61]. miR-340-5p has been shown to upregulate the activity of telomerase and increase cellular telomere length by targeting the telomere protein protection of telomere 1 (POT1) in a murine model of Alzheimer’s disease, which led to an improvement in dementia symptoms [72]. Another study found that miR-185 was negatively associated with POT1, such that overexpression of miR-185 increased telomere dysfunction-induced foci signals in human cancer cells and primary human fibroblasts [74]. Deleting miR-126a induced age-associated telomere shortening, which was associated with inflammation and hepatic dysfunction in a murine model of cholestasis; the authors found that versican was a direct target of miR-340-5p, which mediated these effects [73].

#### 5.1.3. Epigenetic Alterations

Epigenetic changes mediated by changes in DNA methylation, histone post-translational modification and aberrant chromatin remodelling contribute to the pathology of ageing and age-related chronic diseases [61].

Aberrant breast DNA hypermethylation in archived breast tumour tissue has been associated with diminished expression levels of miR-29a, miR-29b, miR-26a, miR-26b, miR-148a and miR-148b. Moreover, microRNA itself can be a target of epigenetic alterations which are clinicopathologically significant. Indeed, the methylation of target microRNAs is thought to contribute to the neoplastic processes of chronic lymphocytic leukaemia and can distinguish chronic lymphocytic leukaemia samples from healthy controls [84]. As a more specific example, hypermethylation of the DNA region encoding miR-148a leads to a repression of this microRNA in pancreatic ductal adenocarcinoma and precursor pancreatic intraepithelial neoplasia lesions [75]; this has been proposed as a biomarker to differentiate pancreatic ductal adenocarcinoma from chronic pancreatitis [75]. More recently, Ma et al. demonstrated that DNA methyltransferase causes the hypermethylation of the promoter region for miR-34a, thereby repressing its expression [85]. This is important as the authors found that increased expression of miR-34a inhibits the activation of the Notch pathway, which improves the sensitivity of pancreatic cancer cells to molecular therapies [85].

The significance of microRNA-mediated changes in DNA hypermethylation extends beyond oncology. The methylation status of miR-7 has been shown to differentiate between different clinical phenotypes of patients with COPD [76]. Hypermethylation was more common in patients with emphysema compared to those who had frequent exacerbations, chronic bronchitis and asthma COPD overlap syndrome [76].

The interconnected relationship between microRNA expression and hypermethylation reflects the reciprocal pathways by which epigenetic alterations cause disease [86]. This can be seen in the mechanisms of chromatin remodelling. Switch/sucrose nonfermentable complexes (SWI/SNF) and imitation switch complexes (ISWI) effect chromatin remodelling and are influenced by microRNA expression. Arid1a is a subunit of the SWI/SNF complex, and Zhu et al. showed that miR-223-3p regulated the expression of Arid1a, which promoted the proliferation and invasion of gastric cancer cells [77]. Similarly, Wang et al. showed that MiR-146b-5p inhibited the SMARCA5 subunit of ISWI in glioma stem-like cells/mesenchymal stem cell fusion cells, which led to an inactivation of a transforming growth factor-β (TGF-β) pathway and impaired proliferation and invasion of the fusion cell, thus suggesting a critical role in malignant progression [87].

#### 5.1.4. Loss of Proteostasis

Ageing and numerous ageing-associated chronic diseases such as Alzheimer’s are associated with the accumulation of pathologically altered proteins due to deficiencies in normal mechanisms of proteostasis [61]. This could be due to disease-specific mutations increasing the risk of protein misfolding, or, alternatively, changes in the pathways for translation of proteins can promote translational errors leading to disease [88]. Impaired proteostasis can also occur secondary to dysfunction of regulatory components which normally remove defunct proteins, such as the unfolded protein response of the endoplasmic reticulum [61]. Additionally, impaired degradation of proteins may be mediated by deficits in pathways such as chaperone-mediated autophagy, which is dependent on factors such as lysosome-associated membrane protein type 2A (LAMP2A) [61].

Researchers are currently exploring the putative effect of microRNAs on cellular proteostasis. Changes in the expression of miR-34 have been shown to affect proteostasis, leading to the accumulation of protein aggregation markers, early ageing and features of neurodegeneration in *Drosophila melanogaster* [78]. This effect was mediated by the dysregulated translation of proteins, with altered H3K27me3 and specific changes affecting the Lst8 subunit of target of rapamycin complex 1 (TORC1), identified as possible targets of miR-34 [78]. In another study, miR-71 was shown to promote ubiquitin-dependent protein turnover [89]. Interestingly the authors found that miR-71 targeted the Toll receptor domain protein TIR-1 in AWC olfactory neurons and that the effect of olfaction on this axis influenced proteostasis and longevity [89]. In laminopathy, dysregulated proteostasis affecting the nuclear matrix protein lamin A is associated with multisystem pathology and syndromes of progeria [90]. This is mediated by a confluence of additional hallmarks including cell senescence, apoptosis and unstable genome integrity [90]. Interestingly, microRNAs have been proposed as potential therapeutic agents for use in laminopathy [90]. Indeed, Nissan et al. showed that miR-9 inhibited the expression of lamin A and the precursor progerin, thus preventing the toxic accumulation of progerin in patients with Hutchinson–Gilford progeria syndrome [79].

Moreover, there is interest in how microRNAs affect specific mechanisms of proteostasis [91]. Researchers are investigating how microRNAs impact the unfolded protein response and what significance this relationship has clinically [92]. Fields et al. showed that miR-320a targets the eukaryotic initiation factor 2 (eIF2) signalling pathway to affect control of the unfolded protein response in colorectal cancer cells [80]. Similarly, microRNAs control chaperone-mediated autophagy; Let7b microRNA increases the expression of LAMP2A, which affects the degradation of the mutant huntingtin aggregates which cause Huntington’s disease [93]. Consequently, let7b microRNA is thought to play an important role in the pathophysiology of Huntington’s disease and has been suggestive as a candidate clinical biomarker for this condition [93].

#### 5.1.5. Disabled Macro-Autophagy

Cytoplasmic material, including nonproteinaceous macromolecules and organelles, is sequestered in autophagosomes which subsequently fuse with lysosomes, leading to the destruction of the contents; dysfunction of this process is implicated in pathological ageing [61]. Researchers are endeavouring to elucidate the mechanisms by which microRNAs may regulate this process and thus contribute to disease [94].

Inhibiting miR-33 in lung macrophages was found to reduce fibrotic changes following bleomycin in a murine model of pulmonary fibrosis [81]. Interestingly, this was associated with upregulated signs of autophagy and mitophagy, which the authors proposed contributed to this therapeutic effect [81]. A separate study found that miR-125b regulated autophagy in patients with thyroid cancer and that decreased the expression of hsa-miR-125b could differentiate malignant from benign thyroid tumours [82]. Here, miR-125b was found to target the mitogen-activated protein kinase (MAPK) and AKT/mechanistic target of rapamycin (mTOR) signalling cascades, which may have mediated the effects of this microRNA on autophagy and resultant carcinogenesis [82]. Furthermore, Ning et al. demonstrated that the expression of miR-494 was upregulated in the myocardium of rats with diabetic cardiomyopathy [83]. By analysing the expression levels of BCl-2 and Bax, the authors showed that miR-494 upregulated autophagy and also regulated phosphorylation of the phosphoinositide 3-kinase (PI3K)/AKT/mTOR signalling pathway, which, the authors argued, provides valuable insights into the aetiological mechanisms driving diabetic cardiomyopathy [83].

### 5.2. Antagonistic Hallmarks

#### 5.2.1. Cellular Senescence

Cellular senescence refers to the stable arrest of proliferation (see Table 2). Senescence occurs in response to tissue damage; it is strongly associated with ageing and occurs in all cell types [61]. This process relies on factors such as the activation of the tumour suppressors CDKN2A/p16 and tumour protein 53 and depletion of lamin B1 from the nuclear envelope [61]. Failure or bypass of senescence is implicated in carcinogenesis, whilst nonproliferative diseases such as pulmonary fibrosis, hepatic steatosis and metabolic syndrome are associated with the senescence-associated secretory phenotype [61]. The senescence-associated secretory phenotype is a heterogenous and complex pro-inflammatory mechanism which is thought to promote localised healing in response to tissue injury: however, this results in disease when it becomes dysregulated [61]. The molecular regulation of senescence pathways is a rapidly evolving area of research, and microRNAs are now understood to have an important role at both the transcriptional and post-transcriptional levels [95].

Indeed, significant research is focused on further understanding the regulatory role of microRNAs in senescence pathways and how this can be applied clinically [95]. Using human cell lines and murine models, Hui et al. showed that the expression of miR-3200-3p in tumour-derived exosomes promotes the senescence of regulatory T cells in non-small-cell lung cancer and potentiates carcinogenesis [96]. This was mediated by decreasing the expression of damage-specific DNA-binding protein 1 which consequently suppressed DCAF1/glutathione S-transferase P1 [96]. MicroRNA regulation of cell senescence has also been implicated in gastric cancer. For example, bromodomain-containing protein 4 (BRD4) has been shown to upregulate the E2F-mediated expression of miR-106b-5p, which suppresses p21 and thereby inhibits cell senescence [97]. Importantly, JQ1 is thought to mitigate the proliferation of gastric cancer cells by inhibiting BRD4 and the E2F/miR-106b-5p/p21 axis and thus inducing senescence [97]. This pathway has been identified as a potential target for future therapeutic targets.

There have been similar findings in nonproliferative chronic diseases. Lu et al. investigated the importance of microRNA regulation of cellular senescence in the pathogenesis of chronic obstructive pulmonary disorder [98]. Overexpression of mir-377-3p was shown to induce senescence in lung fibroblasts, which potentiated pathological changes such as airway remodelling. This occurred secondary to profibrotic effects of the senescence-associated secretory phenotype and suppression of the ZFP36L1 protein [98]. Furthermore, Zhao et al. considered how microRNA and senescence may contribute to age-associated cardiac dysfunction [99]. Firstly, they found that there was an age-associated fall in miR-203 expression which corresponded to increased cardiac dysfunction. The authors showed that miR-203 was important for regulating senescence in cardiomyocytes and, interestingly, old transgenic mice with systemic overexpression of miR-203 exhibited improved cardiac function compared to wild-type controls [99]. The authors demonstrated that this increase in miR-203 attenuated age-associated senescence and remodelling seen in the murine cardiac tissue. This was in part mediated by the actions of miR-203 in suppressing poly (ADP-ribose) polymerase 1, leading to an increase in nicotinamide adenine dinucleotide^+^ and a resultant improvement in mitochondrial function in senescent cardiomyocytes [99]. Similar findings have implicated microRNA regulation of senescence with other chronic conditions, such as Alzheimer’s disease [100].

Other studies are actively exploring how senescence-associated microRNAs can be targeted therapeutically. Zhang et al. found that increased miR-217-5p expression in exosomes secreted by senescent epithelial cells in a murine model was associated with the progression of pulmonary fibrosis following paraquat exposure [101]. The authors found that increased expression of miR-217-5p led to the inhibition of SIRT1, which resulted in increased levels of acetylated β-catenin and the activation of Wnt signalling. Accordingly, increased miR-217-5p expression levels mediated the activation of pulmonary fibroblasts such that suppression of miR-217-5p with an antagomir resulted in a reversal of the paraquat-induced pulmonary fibrosis [101].

Similar therapeutic strategies have been investigated in other studies. A recent study investigated the cartilaginous expression of miR-708-5p, which was shown to decline with age [102]. This was also associated with the development of more severe osteoarthritic lesions in response to mechanical stress in older rats compared to younger subjects. Crucially, the authors showed that local injection of an agonist for miR-708-5p could reduce degenerative changes and partially restore the chondrogenic differentiation capability of the tissue by suppressing TLR4 expression in the Toll-like receptor 4/nuclear factor-κB (NF-κB) pathway [102]. Similarly, dysregulated expression of specific microRNAs and the resultant effects on senescence have been implicated in the aetiology of diabetic ulcers [103]. Wei et al. developed a novel bioactive wound dressing using small extracellular vesicles which exhibited enriched expression levels of MiR-17-5p [104]. By targeting phosphatase and tensin homologue (PTEN) and p21, this was shown to inhibit senescence and promote angiogenesis and collagen deposition, with a resultant enhancement of diabetic wound healing [104].

The development of therapeutic strategies targeting senescence-associated microRNAs illustrates the centrality of these microRNAs to the aetiology of diseases. This may also provide indirect validation for the use of senescence-associated microRNAs to guide prognostic assessments, though more research is needed to confirm this.

**Table 2:** MicroRNAs and the antagonistic hallmarks of ageing.

Hallmark	MicroRNA	Effect	Model	Target/Mechanism	Findings	Reference
**Cellular Senescence**						
	miR-3200-3p	↑	Cellular and murine model	DDB1 in Treg cells	Inhibition of VEGFR2 upregulates miR-3200-3p which targets DDB1 in Treg cells to promote senescence in non-small-cell lung cancer	[96]
	miR-377-3p	↑	Human patients and cellular and murine models	ZFP36L1	miR-377-3p promotes lung fibroblast senescence and suppresses ZFP36L1 to exacerbate COPD	[98]
	miR-106b-5p	↑	Human gastric cancer cell lines and patient gastric tissue samples	E2F/miR-106b-5p/p21 axis	BRD4 modulates the proliferation of gastric cancer cells by controlling cellular senescence by targeting E2F/miR-106b-5p/p21 axis	[97]
**Mitochondrial Dysfunction**						
	miR-128-3p	↓	Murine asthma model	SIX1	miR-128-3p controls airway inflammation by targeting SIX1 and regulating mitochondrial function	[105]
	miR-181a	↓	Murine model	PDCD4	miR-181a targets PDCD4 to modulate mitochondrial fission and apoptosis and preserve left ventricular function following myocardial infarction	[106]
	miR-328-5p	↓	in vitro and murine model	Sirt1	lncRNA Glis2 inhibited miR-328-5p to improve mitochondrial function and mitigate podocyte apoptosis and progression of diabetic nephropathy	[107]
**Deregulated Nutrient-Sensing**						
	miR-221	↑	Breast cancer cell line	PTEN/Akt/mTOR signalling	miR-221 mediated breast cancer cell proliferation and resistance to adriamycin by modulating PTEN/Akt/mTOR signalling	[108]
	miR-125b	↑	Murine model	RRAGD/mTOR/ULK1 pathway	Atherosclerotic progression was associated with reduced autophagy and downregulated expression of miR-125b	[109]
	miR-192-5p	↑	Human patients with NAFLD and murine models	Rictor/Akt/FOX01	Increased expression of miR-192-5p promotes hepatic macrophage activation and disease progression in NAFLD by modulating Rictor/Akt/Fox01 signalling	[110]

↑ denotes positive association between microRNA and disease progression/hallmark of ageing; ↓ denotes negative association between microRNA and disease progression/hallmark of ageing; MiR, microRNA; DDB1, damage-specific DNA-binding protein 1; VEGFR2, vascular endothelial growth factor receptor 2; COPD, chronic obstructive pulmonary disease; BRD4, bromodomain-containing protein 4; SIX1, sine oculis homeobox homologue 1; PDCD4, programmed cell death protein 4; Sirt1, sirtuin1; lncRNA, long noncoding RNA; PTEN, phosphatase and tensin homologue; mTOR, mechanistic target of rapamycin; NAFLD, nonalcoholic fatty liver disease; Rictor, rapamycin-insensitive companion of mammalian target of rapamycin; Fox01, forkhead box transcription factor 01.

#### 5.2.2. Mitochondrial Dysfunction

Mitochondrial dysfunction has been implicated in pathological ageing [61]. Factors such as mutations in mitochondrial DNA and deficiencies in proteostasis which accrue over time contribute to a deterioration in the function of the organelle. Reactive oxygen species or mitochondrial DNA leak out of the mitochondria, which leads to a cascade of adverse physiological effects including inflammation and cell death [61]. The rise in reactive oxygen species is also exacerbated by the inherent decline in antioxidant defence, which is associated with ageing [111]. This results in increased oxidative stress, which has been associated with many of the hallmarks of ageing and is an area of ongoing research [111].

Dysregulated microRNA signalling is now recognised to have a significant impact on mitochondrial functionality, and this relationship is the focus of ongoing research [112]. Liu et al. found that miR-128-3p inhibited sine oculis homeobox homologue 1 (SIX1) in a murine model of asthma [105]. This affected processes of mitochondrial fission and fusion and alleviated mitochondrial dysfunction, which mitigated resultant apoptosis and the progression of airway inflammation. The miR-128-3p/SIX1 axis was thus recognised to be an important mechanism in the pathophysiology of asthma, and the authors posited that it could offer a new therapeutic target [105]. In a different study, Cheng et al. found that changes to miR-29a-3p/DNA methyltransferase 3A (DNMT3A) mediated the beneficial effects of curcumin in a murine model of pulmonary fibrosis by regulating fibrotic pathways and mitochondrial function [113]. Curcumin increased the expression of miR-29a-3p in pulmonary tissue, which led to a reduction in DNMT3A levels. This inhibited extracellular matrix remodelling and, interestingly, attenuated a fibrosis-associated increase in mitochondrial function [113].

Similar findings have been seen in studies involving numerous diseases. miR-181-a was shown to reduce both the size of infarction and the level of ischaemia-induced apoptosis following occlusion of the left-anterior-descending artery in mice and also preserved left ventricular functioning [106]. This was mediated by a direct downregulation of programmed cell death protein 4 and BID, which mitigated the apoptosis and mitochondrial fission occurring as a result of anoxia [106]. Similarly, MiR-124 overexpression was shown to alleviate oxidative stress and resultant apoptosis and improve mitochondrial function in an in vitro model of hypoxic ischaemic brain damage by inhibiting the expression of STAT3 [114]. These findings were corroborated in an in vivo model of hypoxic ischaemic brain damage using rats, and, importantly, treatment with a miR-124 agomir reduced cerebral infarct volume, local oedema and neuronal damage and improved neurological function [114].

Other studies have investigated chronic kidney diseases. Long noncoding RNA (lncRNA) Glis2 was found to inhibit the apoptosis of mouse podocytes in an in vitro model of diabetic nephropathy by ameliorating changes in mitochondrial function and morphology [107]. Significantly, lnc Glis2 was shown to act as a competing endogenous RNA for miR-328-5p, which mediated these beneficial effects; miR-328-5p directly inhibited the expression of Sirt1, which was shown to reduce podocyte apoptosis and improve mitochondrial function [107]. Crucially, the authors demonstrated that inducing lncRNA Glis2 overexpression in diabetic mice attenuated podocyte apoptosis and pathological changes associated with diabetic nephropathy, including podocyte foot process effacement and glomerular basement membrane thickening [107]. This validated the clinical utility of their findings. Indeed, the exosomal delivery of miR-204 was investigated as a novel therapeutic strategy to ameliorate mitochondrial dysfunction in a murine model of diabetic nephropathy [115]. This emphasises the potential therapeutic value of understanding microRNA-mediated regulation of mitochondrial function.

#### 5.2.3. Deregulated Nutrient Sensing

Nutrient-sensing networks mediate the cellular response to nutritional status and physiological stress [61]. This involves insulins and insulin-like growth factors (IGFs) and their tyrosine kinase receptor targets, as well as numerous intracellular cascades. The somatotrophic axis, ALK-dependent signalling and mTOR are all important components of nutrient sensing and have been implicated in both longevity and numerous chronic diseases, including malignancies [61,116].

There is growing appreciation for the importance of microRNA regulation of mTOR for the aetiology of malignancies [117]. In 2016, Zhang et al. showed that miR-147 mitigated the malignant progression of a breast cancer cell line by inhibiting the Akt/mTOR pathway [118]. More recently, Yin et al. found that miR-221 mediated tumour cell proliferation and resistance to adriamycin by inhibiting the expression of PTEN, which upregulated the activation of the Akt/mTOR pathway in a breast cancer cell line [108]. Using renal cancer cell lines, Shen et al. demonstrated that miR-188-5p inhibits the proliferation and invasion of renal cancer cells by downregulating myristoylated alanine-rich C-kinase substrate (MARCKS) expression and inhibiting the AKT/mTOR signalling pathway [119]. Interestingly, in the study, miR-188-5p was then used therapeutically to successfully limit renal cancer growth in an in vivo murine model [119]. Similar findings highlighted the clinical significance of microRNA regulation of mTOR in prostate cancer [120].

Moreover, this pathway is implicated in nonmalignant chronic diseases. An interesting historical study found that inhibiting miR-7a activated mTOR signalling, which enhanced the proliferation of pancreatic islet cells in mice; the authors suggested that targeting the mir-7–mTOR axis may therefore have potential therapeutic value for treating diabetes [121]. In 2023, Ning et al. showed that exposure to high glucose upregulated the expression of miR-494, which led to increased autophagy and apoptosis in cardiomyocytes [83]. Crucially, the increased expression of miR-494 was associated with a decreased phosphorylation of the PI3K/AKT/mTOR pathway, which the authors proposed could potentiate the pathogenesis of diabetic cardiomyopathy [83]. In a separate study, atherosclerotic progression was associated with reduced autophagy and the decreased expression of miR-125b using a murine model [109]. Conversely, overexpression of miR-125b upregulated autophagy by modulating the RRAGD/mTOR/Unc-51-like autophagy, activating the kinase 1 (ULK1) pathway, which led to reduced atherosclerotic changes [109]. Likewise, miR-101 is thought to ameliorate the progression of liver fibrosis by inhibiting the PI3K/Akt/mTOR pathway, whilst the effect of miR-155 in suppressing this signalling pathway is also thought to facilitate the development of epilepsy [122,123].

Indeed, the microRNA regulation of additional nutrient-sensing pathways is similarly important for the aetiology of chronic diseases. Forkhead box transcription factor Os (FoxOs) are important transcription factors in nutrient-sensing pathways [61]; increased expression of miR-192-5p has a pro-inflammatory effect in nonalcoholic steatohepatitis and promotes hepatic macrophage activation and disease progression by targeting the rapamycin-insensitive companion of the mammalian target of rapamycin (Rictor)/Akt/FoxO1 signalling pathway [110]. In a different study, MiR-30a-3p was shown to reduce the proliferation and invasion of hepatocellular carcinoma cells by inhibiting IGF-1, which is an important extracellular ligand for nutrient sensing [61,124]. Interestingly, miR-221 was found to propagate androgenetic alopecia by suppressing IGF-1 expression, which downregulated the MAPK pathway in dermal papilla cells and downregulated the PI3K/AKT pathway in dermal sheath cells [125].

Studies are also investigating how microRNA expression is associated with the sequelae of overnutrition. Overnutrition leads to obesity, which increases the risk of numerous chronic diseases through factors such as pathological adipokine secretion, which potentiates a chronic inflammatory state [126,127]. MicroRNAs are now thought to mediate some of these adverse effects. For example, changes in the expression of MiR-34a in visceral murine adipose tissue induced pro-inflammatory macrophage infiltration and activation and mediated the development of metabolic dysfunction including insulin resistance in response to a high-fat diet [128]. Furthermore, there is additional interest in how changes in microRNA expression are implicated in the therapeutic benefit of dietary interventions such as intermittent fasting, which is an area of emerging interest [129].

### 5.3. Integrative Hallmarks

#### 5.3.1. Stem Cell Exhaustion

Adequate stem cell function is essential for the repair and renewal processes which facilitate cellular response to injury; deterioration in this capacity is implicated in pathological ageing (see Table 3) [61]. Intrinsic to this is cellular reprogramming, wherein cells undergo dedifferentiation and simultaneously experience a physiological “rejuvenation” [61]. As a result, the cells are reset and lose adverse ageing-associated features, which is reflected in changes like the lengthening of telomeres [61]. Transcription factors such as octamer-binding transcription factor 4 (OCT4), sex-determining region Y-box 2 (SOX2), Krüppel-like factor 4 (KLF4) and MYC and forkhead box protein M1 (FOXM1) regulate this process [61].

Studies are exploring how microRNA expression levels affect stem cell function. In 2013, Yu et al. found that miR-141-3p downregulated ZMPSTE24, which led to an accumulation of prelamin A in the nuclear envelope and mediated cellular senescence using a model for ageing human mesenchymal stem cells [130]. More recently, Jani et al. showed that the mir-221/222 cluster promotes haematopoietic stem cell quiescence and multipotency by targeting the Fos/activator protein-1 (AP-1)/immediate early genes (IEG) pathway and reducing granulopoiesis [131]. The authors suggested that this could be used to optimise outcomes following bone marrow transplantation [131]. Interestingly, in a different study, miR-146a was found to regulate the functionality of haematopoietic stem cells as well as their progression through haematopoiesis in response to chronic inflammation in a murine model [132]. Here, Zhao et al. also demonstrated that these effects were mediated by changes in the miR-146a/tumour necrosis factor receptor associated factor 6 (TRAF6)/NF-κB/IL-6 pathway, which the authors suggested may have significant implications for the aetiology of myeloid malignancies [132]. Su et al. showed that miR-31 modulated the IL-34/Janus kinase (JAK)–STAT3 pathway to control the differentiation and functional reserve of satellite cells, thereby affecting the regenerative capacity of skeletal muscle [133]. Fascinating studies are now exploring how the microRNA regulation of stem cell function can be used to develop innovative therapeutic strategies [134,135].

Cellular reprogramming is a fascinating process by which the differentiation of stem cells can be reversed. This is regulated by transcription factors such as Oct3/4, Sox2, c-Myc and Klf4 [136]. MicroRNAs are now understood to have a central role in modulating these processes, and dysregulation of microRNA control of these mechanisms is implicated in chronic diseases including malignancies [137,138]. For example, Xie et al. showed that circRNA vacuole membrane protein 1 (CircVMP1) expression contributed to cisplatin resistance and tumour progression in non-small-cell lung cancer cells by downregulating miR-524-5p, which promoted the expression of methyltransferase 3 N6-adenosine-methyltransferase complex catalytic subunit and SOX2 [139]. Similarly, Chen et al. demonstrated that circRNA derived from the pleiotophin gene (circPTN) downregulates miR-145-5p and miR-330-5p in glioma cells to potentiate proliferation [140]. Interestingly, in the study, miR-145-5p was shown be critical for tumorigenesis by promoting the capacity of glioma cells for self-renewal; this was evidenced by factors such as an increase in stemness markers such as SOX2, SOX9 and Nestin [140]. Indeed, in a separate study, miR-122 was shown to reduce the stemness and chemoresistance of hepatic cancer cells by targeting Wnt/β-catenin signalling [141].

**Table 3:** MicroRNAs and the integrative hallmarks of ageing.

Hallmark	MicroRNA	Effect	Model	Target/Mechanism	Findings	Reference
**Stem Cell Exhaustion**						
	miR-31	↓	Murine model	IL34, JAK-STAT3 signalling	miR-31 modulates IL-34/JAK-STAT3 signalling to determine the differentiation and functional reserve of satellite cells, thus regulating the regenerative capacity of skeletal muscle	[133]
	miR-524-5p	↓	NSCLC cell lines	miR-524-5p-METTL3/SOX2 axis	circVMP1 potentiates NSCLC progression and DDP resistance by modulating miR-524-5p-METTL3/SOX2 axis	[139]
	miR-122	↓	Human hepatic cancer cell line	Wnt/β-catenin	miR-122 reduces the stemness and chemoresistance of hepatic cancer cells by modulating Wnt/β-catenin signalling	[141]
**Altered Intercellular Communication**						
	miR-322-5p	↓	Rat model of myocardial infarction	Smurf2, TGF-β/Smad pathway	miR-322-5p/Smurf2 axis modulates TGF-β/Smad signalling to potentiate myocardial injury following myocardial infarction	[142]
	miR-582-5p	↓	Human NSCLC cell lines	Hippo-YAP/TAZ	miR-582-5p induces tumour-suppressive changes in NSCLC cells by downregulating YAP/TAZ signalling	[143]
	miR-133b	↑	Murine model of atherosclerosis	Notch signalling	MiR-133b exacerbates atherosclerosis by activating Notch signalling	[144]
**Chronic Inflammation**						
	miR-210	↑	Human patients with psoriasis vulgaris	FOXP3	Upregulated miR-210 modulates FOXP3 in CD4^+^ T cells to potentiate immune dysfunction in psoriasis vulgaris	[145]
	miR-181b	↑	Human osteosarcoma tissue samples	Il-1β/NF-κB	IL-1β/NF-κB signalling induces overexpression of miR-181b which promotes the proliferation of osteosarcoma cells	[146]
	miR-29a/29b	↓	Human patients with cirrhosis	N/A	Reduced miR-29a/miR-29b expression was associated with upregulated IL-6 and TNF-α and a more advanced grade of cirrhosis	[147]
**Dysbiosis**						
	miR-582-3p	↑	Patients with NASH and in vitro models	TMBIM1	Gut microbiota affected expression of miR-582-3p which potentiated hepatic fibrosis	[148]
	miR-122	↑	Murine model	N/A	Intestinal flora-produced butyrate downregulates miR-122 expression which ameliorates diet-induced hypercholesterolaemia	[149]
	miR-29a	↓	Murine model	N/A	miR-29a alleviated hepatic steatosis, altered the intestinal flora, reduced inflammation and improved lipid metabolism	[150]

↑ denotes positive association between microRNA and disease progression/hallmark of ageing; ↓ denotes negative association between microRNA and disease progression/hallmark of ageing; miR, microRNA; IL34, interleukin 34; JAK-STAT3, Janus kinase-signal transducer and activator of transcription protein 3; NSCLC, non-small-cell lung cancer; METTL3, methyltransferase 3; SOX2, SRY-box transcription factor 2; circVMP1, circRNA vacuole membrane protein 1; DDP, cisplatin; Smurf2, Smad ubiquitin regulatory factor 2; TGF-β, transforming growth factor-β; YAP, yes-associated protein; TAZ, WW domain-containing transcription regulator protein 1; FOXP3, forkhead box P3; IL-1β, interleukin-1β; NF-κB, nuclear factor- κB; IL-6, interleukin-6; TNF-α, tumour necrosis factor-alpha; NASH, nonalcoholic steatohepatitis; TMBIM1, transmembrane BAX inhibitor motif-containing 1.

#### 5.3.2. Altered Intercellular Communication

The dysregulation of intercellular communication is implicated in the aetiology of ageing and associated chronic diseases [61]. Deficiencies in numerous pathways accrue over time which overlap with several other hallmarks including chronic inflammation, dysbiosis and the senescence-associated secretory phenotype [61].

The microRNA regulation of intercellular communication is an emerging area of study. Specific factors in blood, including eotaxin (CCL11), TGF-β and the complement component 1q (C1q) have been associated with pathological ageing [61]. miR-629-3p has been shown to potentiate the effect of IL-13 in the pathogenesis of asthma by downregulating FOXA2 and phosphorylating PI3K and AKT, with the resultant effect of reducing cell viability, promoting apoptosis and increasing the secretion of inflammatory chemokines such as CCL11 [151].

Similarly, the microRNA regulation of TGF-β has clinicopathologic significance. In a recent clinical cohort study involving 164 patients, miR-29a and miR-29b expression levels could differentiate cirrhotic from healthy patients, and reduced expression was associated with higher-grade cirrhosis [147]. Importantly, reduced miR-29a and miR-29b expression was also associated with an increased serum concentration of the pathological cytokines IL-6, IGF-1 and TGF-β1, which are known to propagate an epithelial–mesenchymal transition [147]. Interestingly, Guo et al. found that overexpression miR-322-5p ameliorated changes in myocardial enzymes, oxidative stress markers, myocardial apoptosis and myocardial function in a murine model of myocardial infarction [142]. This effect was achieved by inhibiting Smad ubiquitin regulatory factor 2 (Smurf2) expression and thereby downregulating the TGF-β/Smad signalling pathway [142].

Dysregulated intercellular communication also features complex remodelling of the extracellular matrix, which potentiates pathological tissue fibrosis [61]. Here, transcription factors such as WW domain-containing transcription regulator protein 1 (TAZ) and yes-associated protein (YAP) upregulate the expression of profibrotic genes such as lysyl oxidase, which ultimately leads to a resultant secretion of matrix metalloproteinases and corresponding upregulation of connected profibrotic pathways including Notch [61]. A fascinating study employing in silico network analysis methods demonstrated that miR-130/131 family members regulated fibrotic pathways across numerous diseases and tissue beds [152]. Moreover, the authors demonstrated in vivo that the YAP/TAZ-miR-130/301 pathway is implicated in human pulmonary and hepatic diseases, including idiopathic pulmonary fibrosis and nonalcoholic steatohepatitis [152]. Interestingly, inhibiting the miR-130/301 family in murine models led to a downregulation of YAP/TAZ signalling and mitigated extracellular remodelling and disease progression of both pulmonary and liver fibrosis [152]. In a separate study, Zhu et al. showed that miR-582-5p downregulates YAP/TAZ signalling via phosphorylation and inhibiting the PIP5KC1 and NCKAP1 actin regulators in non-small-cell lung cancer cells [143]. This resulted in a tumour-suppressive effect characterised by reduced proliferation, upregulated apoptosis, and changes to the cellular cytoskeleton [143].

Similar findings are seen in nonmalignant chronic diseases. Hu et al. used human umbilical vascular endothelial cells to investigate the effect of oxidised low-density lipoproteins and potentially elucidate pathological mechanisms driving the progression of atherosclerosis [153]. Oxidised low-density lipoproteins upregulated the expression of miR-496 in the endothelial cells whilst downregulating the Hippo-YAP/ZAP pathway [153]. This induced apoptosis and dysfunction of the vascular epithelial cells [153]. Indeed, a separate study found that miR-133b overexpression caused the disease progression of atherosclerosis in a murine model by activating the Notch signalling pathway [144]. This illustrates the clinicopathologic significance of microRNA regulation of this pathway.

#### 5.3.3. Chronic Inflammation

Ageing is associated with a chronic increase in inflammation which has been described as “inflammaging”. This is characterised by systemic factors, such as an increase in circulating inflammatory cytokines including Il-6 and Il-1β, and also features a progressive deterioration in immune function [61]. Chronic inflammation can both be exacerbated by and also itself potentiate the other hallmarks of ageing, which is again indicative of the reciprocal pathways by which these mechanisms cause disease [61].

MicroRNA regulatory pathways have been implicated in the aetiology of immune dysfunction and resultant diseases [154]. Indeed, miR-155 has been identified as an important regulator of inflammatory pathways, with dysregulated expression contributing to the pathogenesis of cancers, pulmonary disorders, multiple sclerosis and numerous other inflammatory autoimmune diseases [155,156,157]. More specifically, Zhao et al. found that miR-210 was overexpressed in CD4^+^ T cells isolated from patients with psoriasis vulgaris [145]. This was shown to potentiate pathological inflammatory changes in the T-cell function by inhibiting FOXP3 expression [145]. As well as elucidating the potential role of microRNA-mediated immune cell dysfunction in the aetiology of psoriasis, the authors suggested that this could provide a novel therapeutic target [145].

Many studies have demonstrated that microRNA expression levels have a significant relationship with crucial inflammatory cytokines. This is important in the aetiology of malignancies. For example, Guo et al. recently demonstrated that IL-6 promotes the malignant progression and invasion of epithelial ovarian cancer cells by downregulating miR-200c and Let-7c through changes in the STAT3/hypoxia-inducible factor-1α pathway [158]. In a separate study, Pei et al. found that Il-1β upregulated the expression of miR-181a by activating NF-kB [159]. This downregulated PTEN expression and thereby promoted the proliferation of colorectal cancer cells [159]. Similarly, miR-181b overexpression, as induced by IL-1β/NF-κB signalling, was shown to downregulate the expression of PTEN and thereby promote the proliferation of osteosarcoma cells [146].

MicroRNA also mediates the pathological effects of inflammatory cytokines in autoimmune diseases. An interesting network analysis identified numerous microRNAs and corresponding signalling pathways involving IL-1β and chemokine ligand 8, which were implicated in the pathogenesis of psoriasis [160]. Likewise there is emerging evidence linking this relationship with thyroiditis; Li et al. found that increased expression of miR-146a was associated with increased concentrations of IL-17, IL-23 and IL-6 but decreased concentrations of IL-10 in patients with Hashimoto’s thyroiditis [161]. Moreover, increased expression of miR-146a could predict the occurrence of the disease compared to healthy controls [161]. A separate study demonstrated that miR-195-5p modulated the pathological effects of TNF-α in ulcerative colitis by reducing claudin-2 (CLDN2) expression, controlling the structure of the intestinal tight junctions and thereby reducing the permeability of the intestinal epithelial cells [162].

MicroRNA expression and chronic inflammation are similarly implicated in many other chronic diseases. Indeed, reduced expression of miR-29a and miR-29b has been associated with increased concentration of IL-6 and TNF-α and more advanced grades in patients with cirrhosis [147]. Gao et al. found that miR-20a-5p reduced neuronal damage and apoptosis by targeting STAT3 in murine and cellular models of Parkinson’s disease [163]. Furthermore, miR-20a-5p ameliorated associated inflammatory changes, which was indicated by reduced expression of inflammatory cytokines such as IL-6 [163]. Similarly, microRNAs have been shown to potentiate chronic inflammatory changes in diseases such as asthma and many rheumatological conditions, including rheumatoid arthritis, osteoarthritis and intervertebral disc degeneration [164,165,166,167].

Chronic inflammation due to microRNA dysregulation also contributes to the complications of these chronic diseases. Liechty et al. showed that mir-21 is upregulated in diabetic wounds and induces pro-inflammatory macrophages, thus mediating a chronic inflammatory phenotype [168]. Moreover, the authors showed that hyperglycaemia significantly upregulates Mir-21, which inhibits pTEN and positively regulates PI3K and NOX2, thus leading to the formation of reactive oxygen species [168]. In a separate study, Rawal et al. found that the expression of miR-369-3p is reduced in diabetes-associated atherosclerosis [169]. The authors demonstrated that miR-369-3p reduces the metabolic inflammation induced by oxidised low-density lipoproteins and alleviated associated mitochondrial dysfunction; this effect was mediated by downregulating succinate-GPR91 signalling [169]. Interestingly, the succinate receptor (GPR91) expression was increased in patients with coronary artery disease and diabetes, and the authors showed that antagonising GPR91 signalling offers a promising therapeutic strategy [169]. Indeed, Zhang et al. showed that a pathological increase in myocardial infarction-associated transcript (MIAT) mediates neuronal damage, apoptosis and impaired function in ischaemic stroke by downregulating miR-874-3p, which results in increased inflammatory cytokines including IL1B [170].

#### 5.3.4. Dysbiosis

The gut microbiome is an important component of organismal health. The balance and composition of the intestinal flora affects factors such as immune system development and function and response to intestinal infections and also produces bioactive molecules such as short-chain fatty acids [171]. Accordingly, dysregulation of the gut microbiome, or dysbiosis, has been implicated in the aetiology of numerous chronic diseases [61]. Specifically, the ecological diversity, uniqueness of the microbiome and colonisation status of specific organisms are acknowledged to be important clinicopathologic factors [61]. Investigations have demonstrated the efficacy of interventions such as faecal microbiota transplantation and probiotics which target the gut microbiome; this validates the clinical significance of the gut microbiome and shows how it is becoming an innovative therapeutic target [61].

Research has shown that microRNAs are crucial mediators of host–microbiome interactions [171]. Microbiome composition can affect the expression of circulating microRNAs in the host and thus cause chronic diseases including chronic inflammatory conditions and cancers [172]. Interestingly, it is also thought that microRNAs contained within extracellular vesicles may be released by host intestinal cells to regulate the gut microbiome [173]. As such, faecal microRNAs are thought to have an important role in regulating the gut microbiota [172]. Accordingly, the clinicopathologic significance of the interaction between microRNA expression and the microbiome is an area of ongoing research [174]. Studies have shown that this relationship potentiates the pathogenesis of numerous chronic diseases, including metabolic disorders such as obesity, malignancies, irritable bowel syndrome, inflammatory bowel disease and systemic autoimmune diseases [175,176,177,178,179,180].

More specifically, there is a significant link with inflammatory diseases. A fascinating study found that chlorogenic acid alleviates diabetic symptoms and intestinal inflammation in diabetic mice by upregulating microRNA-129-1-3p and microRNA-666-5p [181]. This promoted the intestinal growth of *Lactobacillus johnsonii*, which mediated these therapeutic effects and demonstrated the clinicopathologic significance of the microRNA–microbiome axis [181]. This effect on local inflammation is naturally important for the development of inflammatory bowel disease. Indeed, Ma et al. demonstrated that pathological changes in the gut microbiota were associated with changes in microRNA expression in a murine model of ulcerative colitis [182]. Moreover, Huang et al. showed that miR-582-3p expression was upregulated in patients with nonalcoholic steatohepatitis [148]. The authors found that inhibiting miR-582-3p attenuated the proliferation of hepatic satellite cells and promoted hepatic satellite cell apoptosis [148]. By downregulating transmembrane BAX inhibitor motif-containing 1 (TMBIM1), miR-582-3p upregulated factors such as α-smooth muscle actin (α-SMA) and Toll-like receptor 4 (TLR4), which are myofibroblast markers, thus potentiating hepatic fibrosis. Crucially, miR-582-3p expression corresponded to changes in gut microbiota, and the authors proposed that intestinal dysbiosis may precipitate dysregulated miR-582-3p expression, thus contributing to the pathogenesis of nonalcoholic steatohepatitis [148].

Similarly, the microRNA–microbiome axis has been implicated in numerous metabolic disorders. Das et al. used a murine model to investigate the clinicopathologic importance of microbiota-associated metabolites [149]. The authors showed that butyrate upregulates ARE/poly(U)-binding/degradation factor-1 (AUF-1) in mice, which inhibits Dicer-1 expression and thus reduces expression of miR-122; this effect ameliorated diet-induced hypercholesterolaemia [149]. Importantly, this butyrate was shown to be produced by the intestinal flora of the mice after the mice were treated with antibiotics and this beneficial effect was removed [149]. Another study showed that miR-29a alleviated hepatic steatosis and inflammatory changes in mice in response to a high-fat diet; overexpression of miR-29a also protected the integrity of the intestinal epithelial barrier from damage induced by the high-fat diet [150]. Interestingly, miR-29a overexpression led to beneficial changes in the intestinal flora which were associated with reduced inflammation and improved lipid metabolism [150].

Innovative studies are further exploring how the microRNA–microbiome axis affects health. Indeed, microRNA are now thought to mediate the pathological effects of intestinal dysbiosis, contributing to the development of neuropsychiatric diseases such as cognitive disturbance, anxiety and depression [183,184,185]. In this way, microRNAs may have a central role in the brain–gut axis. Moreover, there is growing interest in how the microRNA–microbiome axis can be targeted therapeutically, whilst the clinicopathologic significance of faecal microRNAs is also being investigated [186,187].

## 6. Discussion

Multimorbidity is a very common feature of clinical practice; it is associated with adverse outcomes for patients and significant challenges for healthcare providers. Crucially, patients with multimorbidity are routinely excluded from clinical trials, which limits the clinical applicability of their results. As such, there is an urgent need to develop new biomarkers to assess the prognostic burden of multimorbidity. Whilst microRNAs have been shown to predict common clusters of comorbidities, very few studies have considered how microRNA expression relates to the prognostic burden of multimorbidity. The hallmarks of ageing are important factors potentiating the pathogenesis of multimorbidity, and this study highlights the breadth of research showing how microRNAs mediate the pathological effects of these hallmarks in the aetiology of many chronic diseases. Accordingly, this study proposes that the hallmarks of ageing can be used as a framework to develop a panel of microRNAs to assess the prognostic burden of multimorbidity.

This is a novel approach for prognosticating multimorbidity. Existing molecular strategies have focused on using inflammatory or epigenetic markers to characterise pathological ageing [188,189]. Freitas et al. showed that the expression of microRNAs relating to cardiovascular comorbidities were associated with increased severity of obstructive sleep apnoea, but no attempt was made to correlate microRNA expression with the burden of comorbidity [190]. In a fascinating project, Sayed et al. developed an inflammatory ageing clock which uses inflammatory and immunological markers to characterise the physiological age of patients [188]. This model was associated with multimorbidity as assessed by a simple count of diseases. In this study, however, a comprehensive panel of microRNAs was not used, a detailed assessment of how this model relates to the prognostic burden of multimorbidity was not made and this study only used inflammatory and immunological biomarkers, which may overlook other physiological determinants of ageing and multimorbidity. Iannone et al. found that miR-181a was significantly associated with the burden of multimorbidity, as assessed by the CIRS comorbidity score [191]; the authors only investigated this one microRNA, however, which is known to regulate inflammatory molecules.

A separate study identified a panel of microRNAs which could identify high-risk patients as indicated by the haematopoietic cell transplantation comorbidity index. This study used a haematological-specific comorbidity index which limits the wider applicability of the results, and the authors acknowledged the need for more studies to investigate how microRNA can be used to predict mortality and prognosis [192]. Ultimately, much more research is needed to elucidate the potential use of microRNA in assessing the prognostic burden of multimorbidity. In contrast to these previous studies, the present article proposes adopting a multifaceted mechanistic approach to multimorbidity assessment which uses microRNA to provide a comprehensive molecular assessment of the different hallmarks of ageing to assess prognosis. This may broaden the clinical relevance of the model.

Developing a molecular morbidity score using microRNA has many potential applications. Firstly, by providing a summative molecular assessment of the hallmarks of ageing, it would enable research to further elucidate the aetiology of multimorbidity. Specifically, it could be used to explore how differences in the hallmarks of ageing correspond to different multimorbidity phenotypes. Exploring the mechanisms of multimorbidity is an area of ongoing research, and as well as positing clear academic merit, this has the potential to facilitate innovative new therapeutic strategies which target these mechanisms [8]. Indeed, advancements in theoretical understanding of ageing are currently being used to develop drugs which inhibit ageing processes in order to ameliorate multimorbidity [193]. As well as advancing the theoretical understanding of multimorbidity, a molecular morbidity score could also be used to assess the mechanistic effects of interventions as a composite molecular assessment of health.

Secondly, a molecular morbidity score would have clear clinical benefit, as it would enable precise prognostication of patients with multimorbidity. This could be used to identify clinically significant multimorbidity and target specific interventions for high-risk patients [8]. Furthermore, the proposed score would have the potential to guide clinical decision making, including ceiling-of-care decisions and fitness for surgery. As a more theoretical application, it could also provide molecular validation for other methods of prognostication for patients with multimorbidity, such as the syndrome of complex multimorbidity [194]. Similarly, this score could assess the molecular significance of more general prognostic metrics, such as sarcopenia [195].

Crucially, a molecular morbidity score would enable precise and objective characterisation of the burden of multimorbidity. This could facilitate wider integration of older patients with multimorbidity into clinical trials [23]. For example, the proposed molecular morbidity score could be used to randomise patient inclusion, or it could be included as a potential confounding variable in regression analyses. As such, it is potentially transformative.

Nevertheless, significant challenges must be acknowledged. Firstly, microRNA assessments can be expensive, and so the cost-effectiveness of this approach must be considered. Also, there are wider issues facing microRNA research, including lack of standardisation of research methods, which could potentially encumber the development of this idea [196]. A more detailed discussion of the practicalities of using microRNAs as clinical biomarkers has been outlined elsewhere [196,197].

Moreover, specific microRNAs may have many physiological effects, which vary based on tissue location and specific disease factors [198]. These potential effects may even oppose one another, and how this heterogeneity can be accounted for in the proposed molecular morbidity score should be a focus for further study [198]. Additionally, this article included studies using different species and disease models to illustrate the breadth of research linking microRNA expression with the hallmarks of ageing. Whilst admittedly, many of these microRNAs are known to be conserved between species, the actualisation of the proposed molecular morbidity score will require a systematic approach to developing a panel of suitable microRNAs which are clinically applicable for human patients. In these human patients, it will be necessary to consider how additional clinical factors, such as polypharmacy, may affect microRNA expression and thus confound analyses exploring its association with multimorbidity. This should be the focus of further research. Indeed, there is a clear academic and clinical mandate to explore this novel utility further.

Furthermore, the present study proposes the need for a paradigm shift in how ageing and chronic diseases are considered academically. There is a significant focus in the literature about the pathology of ageing [61]. Indeed, the geroscience hypothesis itself proposes that the mechanisms of ageing propagate the pathogenesis of chronic diseases, and there are many studies focused on developing “anti-ageing” medication [199,200]. The connection between ageing and chronic disease may be seen as an associative rather than causative relationship. Indeed, it is accepted that exogenous and endogenous factors contribute to pathological molecular changes that correspond to the hallmarks of ageing. At a single moment, there is a residual risk of exposure to these factors, and once a pathological change has occurred, there is an increased risk of chronic disease. Over time, by statistical inference, an individual is exposed to more factors and so more pathological changes accrue, which leads to an increased risk of disease.

As such, chronic disease may be described as a function of cumulative risk. This “cumulative risk” theory of chronic disease may be seen as a more mechanistic description for the intrinsic association between ageing and chronic disease. Importantly, it avoids pathologizing the process of getting older, which has the risk of stigmatising the elderly. Indeed, viewing ageing as inherently pathological seems like an overly reductionistic view of the human experience, and, especially from a clinician’s perspective, it belies the dignity of old age. The potential merit of a “cumulative risk” approach to chronic disease is an interesting area for further research.

In conclusion, this study proposes using the hallmarks of ageing as a framework for developing a microRNA panel to assess the prognostic burden of multimorbidity. This epitomises translational research and is a fertile and fascinating area for future study. The proposed molecular morbidity score places microRNAs at the furthest frontiers of the clinical science interface as part of an increasingly personalised and integrative approach to clinical practice.
